# Comparative evaluation of clinical performance, child and parental satisfaction of Bioflx, zirconia and stainless steel crowns in pediatric patients

**DOI:** 10.12688/f1000research.133464.2

**Published:** 2023-12-21

**Authors:** Ishani Rahate, Punit Fulzele, Nilima Thosar

**Affiliations:** 1 Department of Pediatric and Preventive Dentistry, Sharad Pawar Dental College, Datta Meghe Institute of Higher Education and Research, Wardha, Maharashtra, 442001, India

**Keywords:** Bioflex crown, zirconia crown, aesthetic, early childhood caries, semi-permanent restorations

## Abstract

**Background:**

Pediatric dental treatment is challenging in patients with early childhood caries. For clinician its difficult to manage child and provide good work at the same time. Its necessity to have the best equipments as well as materials. Nowadays, aesthetics play an important role in managing decayed teeth. Zirconia crown is better option but requires excessive preparation. As Bioflx is newly developed crown and has combined stainless steel and zirconia properties.

**Aim:**

To assess the clinical performance and child and parental satisfaction of Bioflex crowns compared to zirconia and stainless steel crowns.

**Methods:**

In this comparative study of Bioflx crowns with zirconia and stainless steel crowns, children aged three to seven years old will be selected, and 72 primary teeth requiring crowns will be randomly distributed into three groups, n = 24: **Group I:** Preformed stainless steel crown, control; **Group II:** Preformed Bioflex crown; **Group III:** Preformed zirconia crown. Crowns will be evaluated for recurrent caries, plaque accumulation, restoration failure, gingival status opposing tooth wear, and clinicians and parental satisfaction at zero, three, six, and 12 months.

**Results:**

Bioflx crown will have better clinical as well as parental satisfaction among zirconia and stainless steel crowns.

**Conclusions:**

The Bioflx crown can be used as an alternative economical esthetic full-coronal restoration for primary teeth.

**Trial registration:**

CTRI registration number: CTRI/2023/05/052256; Date of registration: May 03, 2023.

**Protocol version:**

Two; Date: April 22, 2023

## Introduction

### Background and rationale

In most preschoolers, early childhood caries pose a significant problem. If left untreated, they can lead to the degeneration of a child’s oral health. ^1^ Managing deciduous, deformed, decayed, or traumatized teeth with tooth-colored restoration is challenging in children because of their miniature tooth size, larger pulpal chamber, thin enamel, and decreased surface area for restoration, accompanied by specific behavior management problems in young pediatric patients. Aesthetic concern plays a vital role in modern dental practice. Understanding the child’s and parental aesthetic perception is necessary for good clinical practice. ^2^^,^^3^

An optimal anterior restoration should have better durability, ease of handling, be aesthetically acceptable, and be cost-effective. There have been many options for full coverage restoration of deciduous teeth, each with technical, functional, or aesthetic limitations. ^3^^,^^4^ The demand for beautiful smiles is increasing among children and adults. A child’s looks can affect their achievement in social acceptance, quality of life, and physical and psychological health. A variety of aesthetic solutions are available, including full-coverage crowns for deciduous anterior teeth, prefabricated primary zirconia crowns, and pre-veneered stainless-steel crowns. There is possibility of increasing in the occlusal vertical dimension after placing crown which may cause temporomandibular dysfunction. In children it is found that occlusal re-equilibration is attained after treatment. ^5^

The primary dentition should be preserved in a non-pathologic and healthy state for the child’s overall well-being. Pediatric dentists have to balance three priorities: the patient’s behavioral management, the conservation of the tooth structure, and the parents’ satisfaction. ^6^ Continually re-evaluating pediatric dental treatment modalities and techniques is necessary because the advancements in dental materials for children over the last few decades have led to constant improvement in dental materials suitable for children. Young patients may not necessarily benefit from a treatment approach that was acceptable in the past. There has been a concerted effort to bring various approaches for full coverage restorations in pediatric dental practice. Every technique and material has its merits and demerits. It is noted that there are numerous possibilities for treating carious teeth in young children, ranging from stainless steel crowns and their modifications to other aesthetic crowns like Bioflx and zirconium crowns, which are becoming more and more popular. Bioflx crowns are flexible, durable, and adaptable. They are available as aesthetic preformed pediatric crowns that offer properties of both stainless steel and zirconia crowns. There is a lack of literary evidence for assessment of the properties of Bioflx crowns and their effect on clinical outcomes and parental satisfaction compared to traditionally available options. Hence, this study plans to assess the clinical performance and child and parental satisfaction for Bioflx, zirconia, and stainless steel crowns in pediatric patients.

### Objectives

The objectives are as follows:

• To evaluate and compare the clinical performance of Bioflx, zirconia, and stainless steel crowns in primary dentition based on recurrent caries, gingival health, restoration failure, plaque accumulation, opposing tooth wear, and clinicians’ satisfaction at zero, three, six and 12 months follow up.
• To assess the child and parental satisfaction of Bioflx, zirconia, and stainless steel crowns in primary dentition at zero, three, six, and 12 months.

## Methods

### Study design

**Figure 1.**
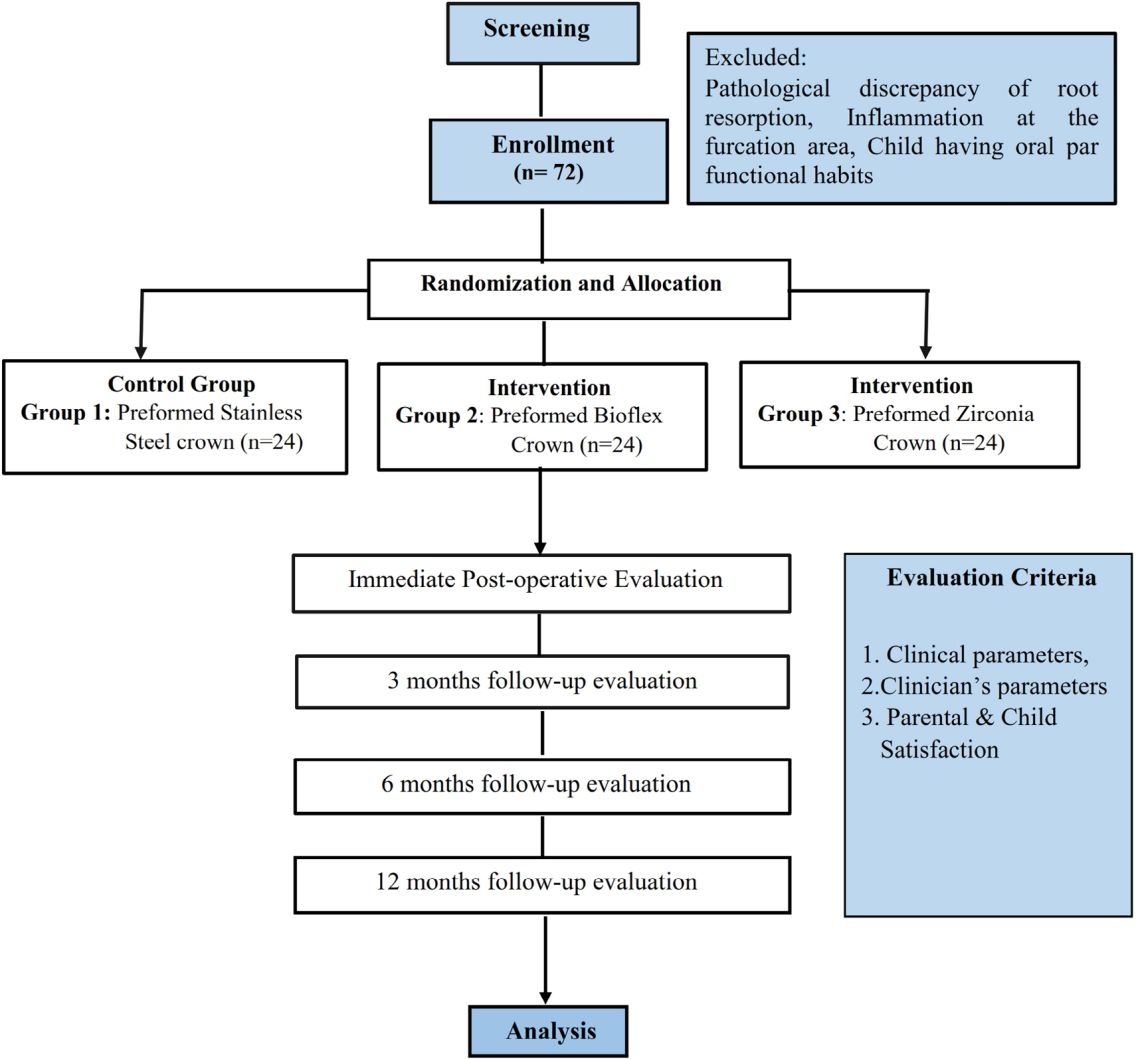
Flow diagram of study participants.

A randomized controlled trial with a parallel group will be the research design for the study. Total 72 primary teeth requiring crowns will be randomly distributed in three groups of 24 each. The allocation will be carried out using computer-generated numbers. After obtaining written informed consent, the subjects will be enrolled, and teeth will be assigned randomly to the groups for receiving the intervention or conventional preformed crowns. The study adheres to the protocol following the Standard Protocol Items: Recommendations for Interventional Trials (SPIRIT) guidelines. ^7^ The allocation of participants and flow diagram for study participants is shown in Figure 1.

### Study setting and population

The present study will be conducted in the Department of Pediatric and Preventive Dentistry of Sharad Pawar Dental College and Hospital, Sawangi (Meghe), Wardha, Maharashtra, India.

In children with more than one tooth requiring a crown, the teeth will be allocated based on random sampling. We will be conducting single blinding in which participants will be blinded. Personal information about potential and enrolled participants will be collected and maintained in order to protect confidentiality before, during, and after the trial. The population of the study is young, healthy children. The research is approved by the institutional ethics committee of Datta Meghe Institute of Higher Education and Research (Deemed to be University) (ref. no: DMIHER (DU)/IEC/2023/565). (IEC) Institutional Ethical Committee Date of approval: February 6, 2023.

### Eligibility criteria

Inclusion criteria ^6^ will be as follows:

• Children aged three to seven years old who are healthy and free of any systemic disease
• Deciduous teeth indicated for crowns
• Deciduous teeth having two-thirds of root structure left radiographically
• Presence of one-third of crown shape

Exclusion criteria ^6^

• A pathological discrepancy of root resorption
• Inflammation at the furcation area
• The child having oral parafunctional habits
• Deciduous teeth having not sufficient crown structure
• Teeth with root caries

Evaluation criteria

The study will be clinically evaluated from the following criteria: crown retention, modified gingival index, plaque index, stain resistance, gingival marginal extension, occlusion, proximal contact, opposing tooth wear, and radiographic assessment.

Clinician’s Satisfaction Criteria will be as follows: surface gloss, roughness, stain resistance, aesthetic, anatomical form, crown fracture, recurrent caries. Parental satisfaction ratings of aesthetic characteristics and their impact of treatment on their children will be evaluated on the basis of size, shape, color and stain using five-point Likert’s scale.

### Sample size

A sample size of children aged three- to seven-years-old will be selected and 72 primary teeth requiring crowns will be randomly distributed in three groups of 24, as follows:

$$N=\frac{{\left(Z_{\left(\alpha /2\right)}+Z_{\beta }\right)}^{2}\left(P_{1}\left(1-P_{1}\right)+P_{2}\left(1-P_{2}\right)\right)}{{\left(P_{2}-P_{1}\right)}^{2}}$$ (1)

$$Z_{\alpha /2}=\text{at 99\% (CI)}=2.576$$

Represents the desired level of statistical significance

$Z_{\beta }=1.28$ : Represents the desired power = 1.28 for 90%

*N* = Minimum samples required for each group

Where,

*P*_1_ = Estimated proportion of study outcome (Child satisfaction % Experimental group) = 99.99% (Approximate to 100%) (1) ^1^

*P*_2_ = Estimated proportion of study outcome (Child satisfaction % Control group) = 53.33% ^1^

At a level of significance at 1% and power 90%

The minimum sample size required:

$$N={\left(2.576+1.28\right)}^{2}\;{\left(0.999\right)}^{*}\;\left(1-0.999\right)+{\left(0.533\right)}^{*}\;\left(1-0.533\right)/0.466=22\;\mathrm{per}\;\text{group}$$

Considering 10% dropout=2

Samples required22+2=24each

$$\text{Total samples required for three groups}=3^{*}N=72\;\text{total}$$

Mathew *et al.,* showed the difference between the two proportions of child satisfaction (%) as the primary variable (1) ^1^

P1(for the experimental group) = 100% (considered as 99.999% approx to 100%) and for the control group (53.333%). We considered a clinically significant margin of difference at 46.666%. Also, we considered the assumption for statistical significance, the highest statistical significance level of 1% alpha value with power (1-beta) at 90%.

We use the test statistics of two independent proportion sample size calculations with the given formula samples for taking the minimum samples required in each group.

Calculated with 22 samples required in each group, adding 10% of dropout = 2. A total of 22 + 2 = 24 samples are required in each group, with a sample size of 72 children distributed in three groups.

### Recruitment and consent

All the healthy children having more than one deciduous tooth decay requiring a crown will be selected for the study. Parents will be oriented on the implementation of the study.

The study protocol will be explained to the participating children and their parents. Further, a written informed consent will be obtained from their parents.

### Participant allocation/randomisation

The participants will be allocated based on the inclusion criteria. They will be randomized to preformed Bioflx crown and preformed zirconia crown as the intervention group or preformed stainless steel crown as the control group by computer-generated numbers. The research co-investigator will carry out data collection, data entry, and data analysis and will be blinded to group allocation.

### Interventions

Total 72 primary teeth requiring crowns will be randomly distributed in three groups of 24 each. The allocation will be carried out using computer-generated numbers. After obtaining written informed consent, the subjects will be enrolled, and teeth will be assigned randomly to the groups for receiving the intervention or conventional preformed crowns.

Group 1: Preformed stainless steel crown: control group

(3M™ ESPE™)

The material for coronal build-up for this group is a preformed stainless-steel crown(the control group). An appropriate-sized preformed stainless-steel crown will be selected. Tooth preparation will be carried out with tapered bur (pyrax Dental Mani Diamond Burs TR-13C) to reduce occlusal surface up to 1 to 1.5 mm. The interproximal reduction will be made mesially and distally. The selected crown size will be checked and a trial fit will be done before cementation. It will require crimping pliers and the crown will be cemented using type 1 glass ionomer cement (GC Gold Label I/ Fuji I). The excess will be removed and proper occlusion will be checked. ^8^

Group 2: Preformed Bioflx crown

The Preformed Bioflx crown ( Kids-e-dental LLP [Limited Liability Partnership]) will be used in this group. A similar sized preformed crown will be selected. Tooth preparation will be carried out with a tapered diamond bur (pyrax Dental Mani Diamond Burs TR [Taper Round] -13C) for occlusal reduction by 1–1.5 mm, including the central groove. The proximal preparation will be around 0.5 mm to clear the contact area. Placement of the crown will be achieved by a snug fit followed by contouring using a How plier (GDC How Plier # Curved (3000/72).

Crown cementation will be carried out using a glass ionomer type I (GC Gold Label I/ Fuji I) and removal of excess cement using floss or explorer.

Group 3: Preformed zirconia crown

The material for crown restoration for this group is the preformed zirconia crown (Kids-e-dental LLP). A diamond bur will reduce the occlusal surface by 1.5–2 mm. Interproximally, contacts will be prepared with a tapered fissure bur. About 1–2 mm subgingival preparation will be performed. The selected crown will be placed and checked. The passive fit of the crown will be assessed and will be luted with dual cure resin cement (Prevest Fusion Ultra D/C / Dual Cure Resin). Consistent firm finger pressure will be applied during cementation. Crown placement will be assessed. ^8^

### Comparison

We will compare the control group (stainless steel crown) with Bioflx and Zirconia crowns on basis of their clinical performance and parental satisfaction.

### Outcome measures

Preformed esthetic crowns and preformed stainless steel crown outcomes will be assessed by the research co-investigator at different time intervals of zero, three months, six months, and 12 months follow-up based on clinical performance, and child and parental satisfaction based on three evaluation criteria, which include clinical, clinicians’ and parental satisfaction. The child and parental satisfaction score will be measured using a questionnaire-based five-point rating Likert’s scale. ^1^ Outcome will be evaluated based on durability, flexibility, self adaptability. Bioflx crowns will be a smart option for pediatric tooth-coloured crowns.

### Data collection

The questionnaire for assessing child and parental satisfaction score consists of four main categories in satisfaction rating of esthetic characteristics. ^1^ These categories are size, shape, color, and stain. The response format will be a five-point Likert scale, ranging from not at all satisfied, with a score of 1, to very much satisfied, with a score of 5.

Another questionnaire scale based on parental ratings of the impact of treatment on their children ^9^ consists of five categories, including 1) The oral health of the child improved after crowns; 2) Parents concern about appearance before crowns; 3) The child avoided smiling before crowns; 4) Child smiling after crowns; 5) Crowns have improved the appearance of the child’s teeth. The response format will be a five-point Likert scale, ranging from not at all (score of 1) to very much (score of 5). ^7^^,^^9^

### Data entry and storage

The research co-investigator will carry out data entry. The principal investigator will review the data entered for discrepancies such as entry errors, enrolment errors, etc. The data entry errors will be checked by a co-investigator by randomly selecting data sheets.

### Data analysis and statistical plan

Statistical analysis will be done using general methodology; continuous variables will be summarized using tables of descriptive statistics: the number of patients with recorded observations, mean, standard deviation, median, minimum, and maximum. Categorical variables will be determined using counts and percentages. Descriptive statistics will be presented by diagnosis and all the results will be calculated using RStudio Version: 2023.03.1+446 (RRID: SCR_000432) will be used. Comparison of continuous parameters between the three groups will be performed using an ANOVA test for quantitative data or Kruskal Wallis test for qualitative data. Categorical variables will be summarized using the frequencies and percentages and compared between the three groups.

### Ethics statement and consent

Ethical approval for the study was obtained from the institutional ethics committee of Datta Meghe Institute of Higher Education and Research (Deemed to be University) (ref. no: DMIHER (DU)/IEC/2023/565); date of approval: February 6, 2023. The trial is registered under the Clinical Trial Registry of India, CTRI registration number: CTRI/2023/05/052256; date of registration: May 03, 2023. A written participant information sheet will be given regarding the details of the study, and it will be explained to participants and their parents before enrolment to the study. Their involvement benefits and harm will be explained to the participants. Written informed consent from the participants will be obtained before involving them in study.

### Confidentiality

Confidentiality of the research data collected will be maintained strictly as per the ethical standards. Only the research assistants and the researchers will have access to the participants’ data in the study.

### Dissemination

Once complete the study will be published in a PubMed, Scopus and indexed journal. The data and results from this study may be presented at conferences and published in scientific journals without revealing the identity of the participants.

### Study status

The study is yet to be started.

## Discussion

The significance of conventional stainless-steel crowns in posterior full coronal restorations in early childhood caries cases are well acknowledged. They have been used for various purposes. To further improvise the longevity and need for better natural-looking restoration in primary teeth, this study plans to compare the Bioflx crown over conventional ones.

Stainless steel crowns have been used for decades and fulfill every aspect of a crown except the aesthetic purpose. As a result, tooth-coloured restorations like zirconia were introduced but they require subgingival preparation, which requires more time and is also not cost-effective for parents. The newly emerging Bioflx crown has super flexibility, is more adaptable, is easy to prepare, and is a faster technique for full coronal restorations in early childhood caries cases. Mathew *et al*. (2020) conducted an *in vivo* study in which bilateral pulp therapy was performed. Patients were divided into two groups of either zirconia or a stainless-steel crown. Patients were evaluated based on gingival inflammation, plaque accumulation opposing tooth wear, and parental satisfaction. They found no statistical difference between the clinical outcome success rates for zirconia and stainless-steel crowns, but less plaque accumulation was noted with zirconia in comparison to stainless-steel. With both the crown types, it seemed that parental satisfaction rates were high. ^1^

Gupta *et al*. (2020) also conducted an *in vivo* study to compare three tooth-coloured crowns and evaluate marginal integrity, surface texture, discoloration, anatomical form, and secondary caries in deciduous anterior teeth over a period of three, six, and nine months. Group I included resin strip crowns, Group II had zirconia crowns, followed by Group III, which had Luxa crowns. They found that the results were statistically non-significant for all parameters except the resin strip crowns, which showed secondary caries and irregular marginal integrity. The zirconia crown showed the best results among the three crowns, followed by the Luxa crown. ^6^

Olegário *et al*. (2021) performed a randomized clinical trial to determine the expectancy of survival rate in one year after endodontic treatment in deciduous molars having restoration with stainless steel and bulk-fill composite crowns. Samples were evaluated and randomized at one, three, six, and 12 months of follow up. The survival rate after one year for the stainless-steel crown was 88% and the bulk fill composite was 75%. In intention-to-treat analysis, the success rate of the bulk fill crown was 86.7% and the stainless steel crown was 82.6%. Both the children and their parents were satisfied with the treatments. ^10^

Another similar study by Murali *et al*. (2022) compared stainless-steel and zirconia crowns. At follow-up, these crowns were evaluated based on proximal contacts, retention, plaque accumulation, marginal integrity, gingival inflammation, and opposing tooth wear. The author concluded that the success rate of zirconia was 93.5% and for stainless steel full coverage restoration was 96.7%. In their statistical analyses, it was observed that there was no significance between the groups, and the preformed stainless steel and zirconia crowns showed good results but zirconia was preferred aesthetically. ^8^

The Bioflx crown has been introduced in pediatric practice as a synthetic crown that will provide better adaptation, durability, and ease of handling with improved aesthetic properties compared to conventional crowns. The limitations of this study will be that long-term follow up will be required for more detailed clinical observation and larger sample size will provide more rigorous results.

The study will help improve the properties of conventional crowns and the Bioflx crown may provide a promising result in terms of the clinician’s and parental satisfaction as aesthetic is a prime concern for parents and children in this era.

## Conclusions

In this protocol, the study will evaluate and compare the clinical performance and child and parental satisfaction for Bioflx, zirconia, and stainless steel crowns in pediatric patients over twelve months. Based on the literature, the preformed stainless steel and zirconia crowns showed good results but zirconia was preferred aesthetically. Similar results are expected in the current study for all groups including Bioflx crowns.

## Data Availability

No data are associated with this article. Zenodo: Extended data for ‘Comparative evaluation of clinical performance, child and parental satisfaction of Bioflx, zirconia and stainless-steel crowns in pediatric patients’, https://doi.org/10.5281/zenodo.7994353. ^7^ Zenodo: SPIRIT checklist for ‘Comparative evaluation of clinical performance, child and parental satisfaction of Bioflx, zirconia and stainless-steel crowns in pediatric patients’, https://doi.org/10.5281/zenodo.7994353. ^7^ Data are available under the terms of the Creative Commons Attribution 4.0 International license (CC-BY 4.0).
