# Supplementary material for: Comparative evaluation of clinical performance, child and parental satisfaction of Bioflx, zirconia and stainless steel crowns in pediatric patients
Source: F1000Res. 2023 Dec 21;12:756. Originally published 2023 Jun 27. [Version 2] doi: 10.12688/f1000research.133464.2 (PMC11190641; doi:10.12688/f1000research.133464.2)
Supplement: Peer review history

# Reviewer report: Reviewer response for version 2

**DOI:** 10.5256/f1000research.158784.r286242

**Published:** 2024-06-20

**Authors:** Ammar T. Kasem

**Affiliations:** 1 Mansoura University, Mansoura, Egypt

1. Authors did not mention the criteria of teeth requiring crown for pediatric teeth.
2. FDI criteria are the most recent and accepted criteria for evaluation of dental restorations (Direct or indirect). It will be better to make a modification of FDI criteria and use them in this study.  
3. How the authors will access the opposing tooth wear? objective or subjective method ?
4. Authors should provide a table including methods to access the evaluation criteria.
5. Authors did not mention how to adjust the occlusion.
6. Authors in this study will use 2 types of cements (GI and resin) which add one more variable. in my opinion authors should use only GI to cement the 3 groups, so that the retention of the crowns in all groups will be accessed.

Is the study design appropriate for the research question?

Yes

Is the rationale for, and objectives of, the study clearly described?

Yes

Are sufficient details of the methods provided to allow replication by others?

Partly

Are the datasets clearly presented in a useable and accessible format?

Partly

Reviewer Expertise:

Fixed prosthodontics and dental materials

I confirm that I have read this submission and believe that I have an appropriate level of expertise to confirm that it is of an acceptable scientific standard, however I have significant reservations, as outlined above.

# Reviewer report: Reviewer response for version 2

**DOI:** 10.5256/f1000research.158784.r232103

**Published:** 2024-02-20

**Authors:** Jayakumar Jayaraman

**Affiliations:** 1 Department of Pediatric Dentistry, Virginia Commonwealth University School of Dentistry, Richmond, USA

I reviewed the article and am satisfied with the revision. I do not have further comments so recommending indexing of this study protocol.

Is the study design appropriate for the research question?

Yes

Is the rationale for, and objectives of, the study clearly described?

Yes

Are sufficient details of the methods provided to allow replication by others?

Yes

Are the datasets clearly presented in a useable and accessible format?

Yes

Reviewer Expertise:

NA

I confirm that I have read this submission and believe that I have an appropriate level of expertise to confirm that it is of an acceptable scientific standard.

# Response

**Published:** 2024-02-22

**Authors:** Ishani Rahate

**Affiliations:** Pediatric Dentistry, Sharad Pawar Dental College, Datta Meghe Institute of Higher Education and Research, Wardha, Maharashtra, India

Thank you for your response and approval.

# Reviewer report: Reviewer response for version 1

**DOI:** 10.5256/f1000research.146456.r189260

**Published:** 2023-09-04

**Authors:** Giuseppe Minervini

**Affiliations:** 1 Multidisciplinary Department of Medical-Surgical and Odontostomatological Specialties, University of Campania, Naples, Italy

The paper has well-designed research methods, appropriate statistical analysis and a relatively good interpretation of the results.

 Please be sure to use only keywords accordingly to medical subject headings (Mesh word) for a better indexing.

 I suggest you add a table with the list of abbreviations used in the text.

 I suggest you implement the abstract in order to make it more understandable to authors.

 The introduction should be expanded perhaps by adding a section on temporomandibular disorders.

 The conclusion is in accordance with the objectives of the research, its results and their interpretation, as well as the relevant literature.

Is the study design appropriate for the research question?

Yes

Is the rationale for, and objectives of, the study clearly described?

Yes

Are sufficient details of the methods provided to allow replication by others?

Yes

Are the datasets clearly presented in a useable and accessible format?

Yes

Reviewer Expertise:

TMD

I confirm that I have read this submission and believe that I have an appropriate level of expertise to confirm that it is of an acceptable scientific standard, however I have significant reservations, as outlined above.

# Reviewer report: Reviewer response for version 1

**DOI:** 10.5256/f1000research.146456.r189258

**Published:** 2023-08-04

**Authors:** Jayakumar Jayaraman

**Affiliations:** 1 Department of Pediatric Dentistry, Virginia Commonwealth University School of Dentistry, Richmond, USA

This study protocol is interesting on a clinically relevant topic. My comments are:

1. Objectives 1 & 2 look similar. I suggest deleting Objective 2.
2. Include manufacturer details of all the products used. For example, Kids-e-dental, Glass ionomer cements etc.
3. How are the examiners trained to place the crowns? What is the education and experience of the providers? Also, who will be conducting the assessment, and how are the examiners calibrated?

Is the study design appropriate for the research question?

Yes

Is the rationale for, and objectives of, the study clearly described?

Yes

Are sufficient details of the methods provided to allow replication by others?

Yes

Are the datasets clearly presented in a useable and accessible format?

Yes

Reviewer Expertise:

Systematic reviews & meta-analysis, clinical trials, pediatric dentistry

I confirm that I have read this submission and believe that I have an appropriate level of expertise to confirm that it is of an acceptable scientific standard, however I have significant reservations, as outlined above.
